# A comparison of endothelial cell count, central corneal thickness and intraocular pressure in different ethnic groups in the Western Cape, South Africa

**DOI:** 10.1007/s10792-025-03426-4

**Published:** 2025-01-31

**Authors:** Raquel Bhika, Moleen Zunza, Derrick Smit

**Affiliations:** 1 Division of Ophthalmology, Faculty of Medicine and Health Sciences, Stellenbosch University, Francie Van Zijl Drive, Parow Valley, 7505 South Africa; 2 Division of Epidemiology and Biostatistics, Faculty of Medicine and Health Sciences, Stellenbosch University, Francie Van Zijl Drive, Parow Valley, 7505 South Africa; 3 Division of Ophthalmology, Room 5053, Clinical Building, Faculty of Medicine and Health Sciences, PO Box 241, Cape Town, 8000 South Africa

**Keywords:** Endothelial cell count, Central corneal thickness, Intraocular pressure, Ethnicity, South Africa

## Abstract

**Purpose:**

To compare endothelial cell counts (ECC), central corneal thickness (CCT) and intraocular pressure (IOP) in different ethnic groups.

**Methods:**

Between January 2019 and December 2021, we enrolled 373 patients who self-identified as native African (116), ethnically diverse (157) or of European descent (100). Mean intraocular pressure (IOP), CCT and ECC were recorded.

**Results:**

IOP was similar between the groups (African mean IOP 15.7 ± 2.2 mmHg, ethnically diverse 15.8 ± 2.2 mmHg, European 16.0 ± 2.0 mmHg, *p* = 0.48). There were age differences but no gender differences between ethnic groups. Mean CCT was 503.6 ± 30.0 µm (African), 516.8 ± 30.0 µm (ethnically diverse) and 539.1 ± 34.2 µm (European) (*p* < 0.002 for all). Mean ECC was 2775 ± 272 cells/mm^2^ (African), 2678 ± 233 cells/mm^2^ (ethnically diverse) and 2639 ± 313 cells/mm^2^ (European). These differences were significant between Africans and Europeans (*p* = 0.001) and Africans and ethnically diverse groups (*p* = 0.01).

**Conclusion:**

ECC was highest in Africans with lowest CCT and conversely, Europeans demonstrated lowest ECC and highest CCT. Ethnically diverse participants demonstrated values between those of Africans and Europeans. We hypothesize that genomic research is required to determine if these differences have a genetic basis.

## Introduction

At birth there are approximately 4000–5000 corneal endothelial cells/mm^2^ in a healthy eye. Over time, there is a gradual decline in the number of endothelial cells in a normal adult eye [1]. The endothelium plays a vital role in maintaining the clarity and transparency of the cornea. An endothelial cell count of 500–1000 endothelial cells/mm^2^ or less indicates that the cornea is at risk of decompensation, stromal oedema and bullous keratopathy [2, 3].

Ophthalmic literature recognizes a correlation between intraocular pressure (IOP) and central corneal thickness (CCT). In a study from Japan, Nomura and coworkers demonstrated a positive correlation between CCT and intraocular pressure in both males (r = 0.44, p < 0.001) and females (r = 0.48, *p* < 0.001) [4]. The literature further suggests that ethnicity may influence this relationship [5, 6]. In the Barbados Eye Studies, Nemesure and coworkers found that black participants had thinner corneas (mean CCT = 529.8 µm) than mixed black and white (mean CCT = 537.8 µm) and white (mean CCT = 545.2 µm) participants [6]. In an earlier publication, we demonstrated that the mean CCT values in African participants, ethnically diverse participants and participants of European descent were 514.77 ± 31.86, 531.77 ± 35.17 and 549.97 ± 30.51 µm respectively. We also found a strongly positive correlation between CCT and IOP (β = 0.021; *p* < 0.001) [5].

Ophthalmic literature also recognizes a correlation between endothelial cell count (ECC) and CCT. In 211 patients from Lithuania (54.03% female), Galgauskas and coworkers found a direct correlation between ECC and CCT (r = 0.232, *p* < 0.01). Furthermore, the literature suggests that the endothelial cell count is also similarly influenced by ethnicity [7, 8]. Matsuda and coworkers compared ECC in age-matched populations of American and Japanese participants and demonstrated a significantly higher ECC in the Japanese participants [8].

In summary, the literature therefore recognizes correlations between IOP and CCT as well as ECC and CCT and suggests that ethnicity may influence these relationships. After considering these findings, we were interested to determine how EEC, CCT and IOP compared between different ethnic groups in our local population in the Western Cape, South Africa. The rationale for this question was the fact that the South African population, and specifically that of the Western Cape province, presents a unique and genetically diverse ethnicity. In Cape Town we predominantly see patients of African descent, patients of European descent and patients of ethnically diverse descent. The ethnically diverse group is a result of South Africa’s complex history and its geographical location who self-identify as a distinct group in the country. In 2010, a genome wide analysis was conducted in the South African ethnically diverse population and found predominantly Khoisan, European, African and to a lesser extent Asian (Indian) genes [9].

This study set out to compare endothelial cell counts, intraocular pressure and central corneal thickness between our different local ethnic groups. To date no study has compared all three variables in various ethnic groups in Southern Africa. This study would also provide important normative data regarding the endothelial cell counts in the African, ethnically diverse and European populations in the Western Cape in South Africa.

## Aims and objectives

Based on earlier research by our group and the research outlined above, we hypothesized that a higher ECC could represent a more efficient mechanism for keeping the cornea relatively dehydrated which, in turn, could translate into lower CCT [5]. The aim of the study, therefore, was to compare the CCT, IOP and ECC between the different ethnic groups in our location. The primary objective of this study was to measure the endothelial cell count in self-reported African, ethnically diverse and European ethnic groups. The secondary objective was to measure the intraocular pressure and central corneal thickness in self-reported African, ethnically diverse and European ethnic groups and to compare these findings with measured endothelial cell counts.

## Methods

### Design

An analytical, cross-sectional study was conducted. The study was approved by the Health Research Ethics Committee at Stellenbosch University and adhered to the ethical guidelines and principles of the international Declaration of Helsinki, South African Guidelines for Good Clinical Practice and the Medical Research Council (MRC) Ethical Research Guidelines. Health Research Ethics Committee Reference number: S18/10/277. Data collection took place prospectively from January 2019 until December 2021 at the Eye Clinic of Tygerberg Hospital in Cape Town, South Africa. Informed written consent was obtained and participation was voluntary. An observer administered questionnaire was completed. These questionnaires obtained demographic data (including sex assigned at birth), information regarding medical conditions and the use of systemic, inhaled or topical corticosteroids. Ethnicity was self-reported. Each participant underwent routine slit lamp examination. Thereafter, three non-invasive special investigations were performed on all eyes considered to be clinically normal and that met the inclusion criteria. In other words, if both eyes were considered clinically normal then both eyes were included and not just the right or left eye of an individual. Mean IOP readings were obtained by calculating the mean of six consecutive intraocular pressure readings. The IOP was measured using an iCare PRO tonometer (iCare, Vantaa, Finland) and readings were only accepted if they registered a green light on the first attempt. Central corneal thickness was measured using the Oculus Pentacam^®^ HR (Oculus Wetzlar, Germany) and readings were only accepted if the quality specification of the Pentacam registered an “OK” response. Endothelial cell count was determined using a Nidek CEM-530 Specular microscope (Nidek, Japan) and readings were only accepted if the specular microscope automatically initiated the scan and an automatic data analysis was obtained. No manual analyses were performed. The IOP readings with the iCare PRO tonometer were taken first to confirm IOP in the normal range set at 10–21 mmHg for this study. These readings were taken sequentially, typically in under 30 s, and the mean value recorded in a Microsoft Office Excel spreadsheet (Microsoft, Washington, USA) by RB. All data were collected by the same person (RB) and all data were entered into the spreadsheet by the same person who was also responsible for forwarding the data to the statistician (MZ). The accuracy of the iCare PRO is given as ± 1.2 mmHg (≤ 20 mmHg) and the repeatability has a coefficient of variation < 8%. The Pentacam readings were taken next and the Pentacam^®^ HR claims both precision and reproducibility of ± 0.1D. Lastly, the specular microscopy was performed on the Nidek CEM-530. All the above measurements took place during one visit lasting no longer than 20 min and no follow up visits were required for the purpose of the study. All visits took place between 14h00 and 15h30 in the afternoon as the clinic is too busy in the morning to gain access to the measuring instruments.

### Selection criteria

South African citizens older than 18 years with clinically normal eyes were invited to participate. Patients with uveitis, glaucoma, ocular hypertension, history of corticosteroid use, corneal pathology and previous intraocular trauma or surgery as well as those who wore contact lenses or were younger than 18 years of age were excluded.

### Sampling and statistical analysis

Participants were selected using convenient sampling. Sample size for one-way analysis of variance (ANOVA) test of CCT was estimated using Stata 17 (College Station, Texas 77,845 USA). Sample sizes of 115 participants were required for each of the three ethnic groups whose IOP, CCT and endothelial cell count means were to be compared. A total sample of 345 participants was required to achieve 90% power to detect differences among the means versus the alternative of equal means using an F test with a 0.05 significance level. The size of the variation in the means was represented by their variance of 0.22. The common standard deviation within a group was assumed to be 6.

Continuous variables were summarized using mean (standard deviation) and categorical variables using count (percent). Chi-squared testing was used to assess the association between categorical variables. Age and ethnic group means were compared using ANOVA. More specifically, ANOVA was conducted to compare IOP, ECC and CCT means between the three ethnic groups, adjusting for age differences. If the ANOVA *p*-value was < 0.05, we conducted pairwise comparison of IOP, ECC and CCT means between ethnic groups using the Tukey method. In general, statistical significance was set at *p* < 0.05. Pearson correlation analysis was used to test the linear correlation between the continuous variables.

All patients, accompanying persons, medical students, nursing students and/or staff members at Tygerberg Academic Hospital (older than 18 years of age) who met the selection criteria were invited to be included in the study.

## Results

A total of 373 participants and 737 eyes were enrolled in this study. The demographics of the participants are shown in Table 1. Most of the participants were female (71.8%) and were originally from the Western Cape province (61.4%) followed by the Eastern Cape (21.2%). Of all the participants included in the study, 9.9% were known with diabetes and 19.3% had hypertension.

**Table 1:** Demographics of participants

Variable	Total (n = 373)	African (n = 116)	Ethnically diverse (n = 157)	European (n = 100)	*p*-value
Age in years (± SD)	41.3 (± 14.8)	36.1 (± 11.4)	43.9 (± 14.2)	43.5 (± 17.6)	< 0.001*
Female sex (%)	268 (71.8)	87 (75.0)	109 (69.4)	72 (72.0)	0.599
*Province of origin (%)*					
Western Cape	229 (61.4)	29 (25.0)	147 (93.6)	53 (53.0)	
Eastern Cape	79 (21.2)	65 (56.0)	3 (1.9)	11 (11.0)	
Gauteng	27 (7.2)	7 (6.0)	1 (0.6)	19 (19.0)	
KwaZulu Natal	13 (3.5)	8 (6.9)	1 (0.6)	4 (4.0)	
Mpumalanga	9 (2.4)	4 (3.4)	0 (0.0)	5 (5.0)	
Northern Cape	8 (2.1)	0 (0.0)	4 (2.6)	4 (4.0)	
Free State	5 (1.3)	1 (0.9)	1 (0.6)	3 (3.0)	
Limpopo	2 (0.5)	2 (1.7)	0 (0.0)	0 (0.0)	
North West	1 (0.3)	0 (0.0)	0 (0.0)	1 (1.0)	

*p values < 0.001 between African and European group and between the African and ethnically diverse group, but not between the European and ethnically diverse population (*p* = 1.0)

As mentioned earlier, ANOVA was conducted to compare mean IOP, ECC and CCT between the three ethnic groups after adjusting for age differences. Only if the ANOVA *p*-value was < 0.05 did we conduct pairwise comparison of mean IOP, ECC and CCT between ethnic groups using the Tukey method. Table 2 presents the predicted marginal means for each ethnic group that were derived using the Tukey method.

**Table 2:** IOP, CCT and endothelial cell count in the various ethnic groups, adjusted for age differences

Variable	African (A)	Ethnically diverse (ED)	A versus ED *p*-value	European (E)	A versus E *p*-value	ED versus E *p*-value	ANOVA *p*-value
IOP (mmHg) (± SE)	17.5 (0.25)	16.9 (0.2)		16.3 (0.2)			0.5
CCT (µm) (± SE)	502.3 (3.7)	517.1 (3.1)	0.001	539.2 (3.5)	< 0.001	< 0.001	< 0.001
ECC (cells/mm^2^) (± SE)	2703.5 (29.0)	2658.0 (24.4)		2616.8 (27.8)			0.07

SE = standard error

The mean age-adjusted CCT differed significantly between the various ethnic groups (*p* < 0.001). However, neither the age-adjusted IOP nor ECC differed. There was a significant difference in the age of the African and European population (*p* = 0.001) as well as between the African and ethnically diverse population (*p* < 0.001), but not between the European and ethnically diverse population (*p* = 1.0).

The Pearson correlation analysis showed no correlation between the means of ECC vs CCT and ECC vs IOP (ECC vs CCT, r = 0.006, *p* = 0.9; ECC vs IOP, r = 0.02, *p* = 0.7) but it did reveal a positive correlation between mean IOP vs CCT (r = 0.3, *p* < 0.001).

## Discussion

According to our knowledge, at the time of submission, this is the only study comparing all three variables (IOP, CCT and ECC) in different ethnic groups in Southern Africa.

In this cross-sectional study, it was found that the IOP did not differ between the 3 groups studied and this correlated with findings of an earlier study conducted in the same setting [5]. This finding was opposite to trends in previous ophthalmic literature indicating that thinner corneas are associated with lower IOPs in the African American population and thicker corneas were associated with higher IOP in the European populations [10, 11]. For example, La Rosa and coworkers demonstrated that the CCT of all African American patients was lower than that of all Caucasian patients, regardless of whether glaucoma was present or not [11]. They concluded that these findings may lead to lower IOP readings in African Americans compared to Caucasians but our findings in both studies did not support this. In the Ocular Hypertension Treatment Study, Brandt and coworkers found that mean CCT for African American subjects was 23 $\mu$m less than for white subjects (*p* < 0.0001) and our findings support this [10]. In the Barbados Eye studies, black participants had thinner corneas (mean CCT ± SD = 529.8 ± 37.7 µm) but higher IOP (mean IOP ± SD = 16.7 ± 4.0 mmHg) while European participants had the thickest corneas (545.2 ± 45.7 µm) and the lowest IOP (14.6 ± 3.0 mmHg). This finding was consistent with our data even though it should be noted that the Barbados Eye studies included 2120 eyes of black participants and only 50 eyes of white participants so those values should be interpreted with caution while our subgroup sizes were much more similar.

Only two other studies have been conducted in South Africa that evaluated the ethnic influences on IOP and CCT [5, 12]. In the South African Eye Study (SAES) with 402 participants (158 African, 177 ethnically diverse and 67 European participants), the African population had the highest mean IOP (15.51 ± 2.49 mmHg) when compared to the ethnically diverse (15.09 ± 2.12 mmHg) and Europeans (15.13 ± 2.53 mmHg) (*p* = 0.07). The CCT in the African population was 514.77 ± 31.86 µm compared to the ethnically diverse 531.77 ± 35.17 µm and European populations 549.97 ± 30.51 µm. Quite unsurprisingly, this finding was consistent with our study since the studies were conducted in the same Eye clinic.

The other study conducted in South Africa noted ethnic variations in IOP and CCT between the African and Indian population in KwaZulu-Natal [12]. Two hundred participants were included, 100 African and 100 Indian of similar ages. The mean CCT was 526.5 ± 37.2 µm for the Indian population and 512.4 ± 38.9 µm for the African population. The mean IOP was 15.3 mmHg in the Indian population and 13.8 mmHg in the African population. (*p* = 0.01) There was a positive but inconsistent correlation between the IOP and the CCT because this was noted in the Indian population (*p* < 0.001) and the total population (*p* < 0.001) but not the African population (*p* = 0.05). This study also noted that the CCT in the local African population was lower than the CCT compared to other African populations in the world and the CCT of the Indian population was lower when compared to other Asian populations [12]. This showcases the unique population of South Africa. In our study, endothelial cell counts differed between the African (2775 ± 272 cells/mm^2^) and European (2639 ± 313 cells/mm^2^) (*P* = 0.001) and between the African and ethnically diverse population (2678 ± 233 cells/mm^2^) (*p* = 0.01). However, once the mean ECC was adjusted for age differences, the mean ECC no longer differed between the three groups (Table 2). Given that the mean age in the ethnically diverse and European groups was significantly higher than in the African group, it is likely that the differences in ECC are probably due to age-related decrease in ECC rather than intrinsic differences between the groups.

At first glance, our initial finding, before adjusting for age differences, correlates with other studies that demonstrated ethnic differences in the endothelial cell counts [13–15]. In the study by Mohd Salih, the mean ECC in a Malay population was 2648 ± 310 cells/mm^2^[13], whereas the mean ECC in a Turkish cohort was reported as 2732 ± 305 cells/mm^2^ by Duman and coworkers [14]. However, the mean age in the Malay population was 45.8 ± 20.7 years (range, 20–87 years) whereas the mean age in the Turkish cohort was 42 ± 17.1 years (range, 6–85 years) so it is again possible that the difference in ECC could be at least partially ascribed to age differences rather than other factors. The same possibility exists in the study where Ewete and coworkers reported that the mean ECC in 359 eyes of 201 healthy African volunteers in Nigeria was 2610.26 ± 371.87 cells/mm^2^ which is more than 100 cells/mm^2^ less than in the South African population [7]. Again, the mean age of the Nigerian population was 50.35 ± 20.13 years (range, 20–93 years) which is much higher than the overall mean age of 41.3 ± 14.8 years in our study population.

The findings in our study suggest that a thinner cornea is associated with higher ECC. Given the fact that the main function of the corneal endothelium is to transport water from the corneal stroma into the anterior chamber to regulate and maintain the hydration of the cornea, it stands to reason that a higher ECC could be responsible for more effective dehydration of the cornea resulting in a lower CCT.

Previous studies have compared CCT, IOP and endothelial cell counts in very distinct ethnic populations with very different genetic characteristics, some in entirely different continents. CCT is highly heritable with a strong genetic association [18, 19]. For example, a study conducted in China demonstrated Hub genes located in the genetic network of the CCT [20]. In a study comparing ECC in 66 eyes of 33 monozygotic twins with 48 eyes of 24 dizygotic pairs, ECC was also found to be highly heritable (82.0%, 95% CI 70.0–92.0%) [21].

What makes our study unique, is that it compares those variables within different ethnic groups within the South African population. The data show that the CCT, IOP and ECC of the ethnically diverse group are consistently between those of the African and European populations. This finding could therefore possibly be explained by the blending of the genes within this ethnic group and this paves the way for further studies to identify specific genes and/or pathways coding for ECC within not only these ethnic groups in the South African population but also others elsewhere. If these genes and/or pathways could be identified it might pave the way for future genetic interventions to slow down or reverse endothelial cell loss in diseases such Fuchs’ endothelial dystrophy and pseudophakic bullous keratopathy.

### Study limitations and strengths

The sample was not randomly selected. The study was conducted in the Western Cape province, and one could argue whether the findings could be extrapolated to the rest of the South African population.

Only 100 European participants were included instead of the intended 115 participants but the difference in the number was small, and the power of the study not affected. The significant difference in age noted between the African and European population and between the African and Mixed population could possibly have influenced our results.

This prospective study was the first of its kind in Southern Africa and was adequately powered to meet its aims and objectives. Ethnicity was self-reported which is important in the study setting.

## Conclusion

This study has compared IOP, ECC and CCT in 3 different ethnic groups. Since both CCT and ECC are highly heritable, future genetic studies may be needed to identify genes and/or other pathways that regulate ECC as these may ultimately provide insight into how endothelial cell loss might be reduced. Those findings may also improve our understanding of the association between ethnicity, CCT, family history and the risk of developing certain types of glaucoma.

## Data Availability

Upon reasonable request data can be requested from the corresponding author.
